# Fabrication and Characterization of Nanocomposite Hydrogel Based on Alginate/Nano-Hydroxyapatite Loaded with *Linum usitatissimum* Extract as a Bone Tissue Engineering Scaffold

**DOI:** 10.3390/md20010020

**Published:** 2021-12-23

**Authors:** Mahnaz Mohammadpour, Hadi Samadian, Nader Moradi, Zhila Izadi, Mahdieh Eftekhari, Masoud Hamidi, Amin Shavandi, Anthony Quéro, Emmanuel Petit, Cédric Delattre, Redouan Elboutachfaiti

**Affiliations:** 1 Department of Chemistry, Faculty of Sciences, Tarbiat Modares University, P.O. Box 14115-111, Tehran 6715847141, Iran; mahnazmohammadpour@modares.ac.ir; 2 Pharmaceutical Sciences Research Center, Health Institute, Kermanshah University of Medical Sciences, Kermanshah 6734667149, Iran; izadi_zh@razi.tums.ac.ir (Z.I.); mahdieh.eftekhari@kums.ac.ir (M.E.); 3 Student’s Research Committee, School of Pharmacy, Kermanshah University of Medical Sciences, Kermanshah 6714415153, Iran; nadermoradi19961@gmail.com; 4 BioMatter-Biomass Transformation Lab (BTL), École Polytechnique de Bruxelles, Université Libre de Bruxelles, Avenue F.D. Roosevelt, 50-CP 165/61, 1050 Brussels, Belgium; Masoud.Hamidi@ulb.ac.be (M.H.); amin.shavandi@ulb.ac.be (A.S.); 5 UMRT INRAE 1158 BioEcoAgro, Laboratoire BIOPI, University Institute of Technology, University of Picardie Jules Verne, 80000 Amiens, France; anthony.quero@u-picardie.fr (A.Q.); emmanuel.petit@u-picardie.fr (E.P.); redouan.elboutachfaiti@u-picardie.fr (R.E.); 6 Université Clermont Auvergne, Clermont Auvergne INP, CNRS, Institut Pascal, 63000 Clermont-Ferrand, France; 7 Institut Universitaire de France (IUF), 1 Rue Descartes, 75005 Paris, France

**Keywords:** tissue engineering, alginate hydrogel, nanocomposite, nano-hydroxyapatite, *Linum usitatissimum* phenolics extract

## Abstract

In the current paper, we fabricated, characterized, and applied nanocomposite hydrogel based on alginate (Alg) and nano-hydroxyapatite (nHA) loaded with phenolic purified extracts from the aerial part of *Linum usitatissimum* (LOH) as the bone tissue engineering scaffold. nHA was synthesized based on the wet chemical technique/precipitation reaction and incorporated into Alg hydrogel as the filler via physical cross-linking. The characterizations (SEM, DLS, and Zeta potential) revealed that the synthesized nHA possess a plate-like shape with nanometric dimensions. The fabricated nanocomposite has a porous architecture with interconnected pores. The average pore size was in the range of 100–200 µm and the porosity range of 80–90%. The LOH release measurement showed that about 90% of the loaded drug was released within 12 h followed by a sustained release over 48 h. The in vitro assessments showed that the nanocomposite possesses significant antioxidant activity promoting bone regeneration. The hemolysis induction measurement showed that the nanocomposites were hemocompatible with negligible hemolysis induction. The cell viability/proliferation confirmed the biocompatibility of the nanocomposites, which induced proliferative effects in a dose-dependent manner. This study revealed the fabricated nanocomposites are bioactive and osteoactive applicable for bone tissue engineering applications.

## 1. Introduction

Bone fracture is a common condition that everyone may encounter in their life. In small fractures, the bone tissue can regenerate by itself, but in large defects, it is required to be treated with proper interventions to improve and help the healing process [1]. Autograft is the gold standard clinical treatment for large bone defects, and despite its acceptable treatment outcomes, suffers from critical shortcomings related to the harvesting process. For instance, we can cite the potential for donor site infection, limited quantity, increased blood loss, donor site pain, hypersensitivity or morbidity, and additional surgical procedures [2,3,4]. Novel treatment strategies have been trying to propose innovative concepts to eliminate the limitations of the current treatment modalities. Alternatively, nanotechnology in combination with tissue engineering has evolved as an innovative concept to bypass the need for autograft and propose sophisticated structures as bone healing materials [5,6].

Scaffolds have central roles in tissue engineering strategies that support cell attachment, migration, growth, and even differentiation. Resemblance to the native structure of bone is the prerequisite and vital for bone tissue engineering. Bone is a biocomposite of collagen and hydroxyapatite (HA) ceramics, where the first one provides the 3D structure, flexibility, and resorbability and the ceramics impart strength and osteoconductivity. Accordingly, 3D nanocomposites containing HA have grabbed significant attention in the bone tissue engineering concept [7,8,9]. Various types of natural and synthetic polymers have been applied to fabricate 3D scaffolds for bone tissue engineering. Natural polymers have fascinating biological properties over synthetic ones, while synthetic polymers have fascinating mechanical properties. Alginate (Alg) is a natural anionic polysaccharide that obtains from brown macro-algae. Due to its biocompatibility, such as easy to process, gel-forming ability, biodegradability, and non-toxicity, Alg has been widely applied in different fields of biomedicine, ranging from drug delivery to regenerative medicine [10]. Using Alg, it is possible to synthesize a 3D hydrogel scaffold for bone tissue engineering. Studies on the use of alginate in bone regeneration have shown its potential in bone tissue engineering applications. However, high hydrophilicity of alginate decreases cell adhesion and protein adsorption; research by Tohamy et al. and Purohit et al. showed that the fabrication of Alg-based nanocomposites with nHA or GO promotes the mechanical properties, bioactivity, protein absorption, and proliferation and adhesion of MG-63 cells [11,12,13].

In addition to structural properties, a sophisticated scaffold should have biological activities. Antioxidant natural substances have shown promising results in accelerating the tissue regeneration process. Natural substances obtained from herbal plants are rich in various bioactive phytochemicals with a wide range of biological properties. The application of these phytochemicals has resulted in effective and accelerated healing outcomes [14,15,16]. Flax (*Linum usitatissimum* L.) is a dicotyledonous plant widely distributed in temperate climate zones, and cultivated as a source of fiber, oil, and medicinal compounds [17]. Flaxseed is an oilseed with various fascinating biological activities that are widely used in industrial and natural health products. It is a good source of polysaccharides, cyanogenic glycosides, cyclic peptides, linolenic acid, cyclic peptides, and alkaloids [17,18]. Flaxseed extracts have various biological activates, such as antioxidant and anti-inflammatory activities beneficial for tissue engineering applications [19]. Nevertheless, the phytochemical composition of aerial parts (leaves and stems) and roots of flax has been less studied compared to the compounds obtained from the flaxseed. Recent years have seen a resurgence of interest for the study of secondary metabolites synthesized from the phenylpropanoid pathway in flax. A group of secondary metabolites called phenolic compounds, which are also present in flax, play a variety of functions, such as in plant growth, developmental processes, and defense responses to stress environments [20,21,22,23]. In fact, several studies have proved that they possess antioxidant and radical scavenging properties [24]. Moreover, some of these products or their components possess antimicrobial properties [25] and exhibit multidirectional phytotherapeutic activity. They have significant role in management of various human chronic diseases, such as cancer, diabetes, and cardiovascular disorders [26]. Furthermore, our laboratory has recently identified the presence of flavones *C*-glycosides in a polar extract obtained from the aerial parts of flax [27]. It has been reported that flavonoids exhibit health-protecting activities because of their strong antioxidant properties. According to several authors, some of these flavonoids have been reported to exhibit various biological activities including anti-inflammatory, anti-viral, anti-fungal, and anti-bacterial activities [28]. Thanks to these favorable properties, flavonoids play an important role in bone development, remodeling, repair, and regeneration [15,29].

Accordingly, in the current study, we fabricated a 3D nanocomposite scaffold based on Alg/nHAp and hydromethanolic extract of aerial parts of flax rich in phenolic compounds and flavonoids as the bone tissue engineering scaffold.

## 2. Results

### 2.1. Extraction and Characterization of Purified Extract from Aerial Part of Linumusitatisimum

In this study, the preparation of aerial part of flax crude extract was realized by hydromethanolic extraction, which was selected to extract organic substances, including compounds of medium and high polarity (e.g., phenolics acids, flavonoid glycosides, etc.).

In most cases, purification of plant crude extracts containing phenolic compounds is necessary because considerable amounts of other compounds may also be extracted. Adsorption on macroporous resins was tested as a means of concentrating and pre-purifying the phenolic compounds present in the crude extract. This method consisted of two steps, including adsorption of the phenolic compounds (due to the strong noncovalent bonding and aromatic stacking interactions) from aqueous solution obtained from crude extract, followed by desorption of the targeted phenolic compounds with ethanol. Water-soluble impurities were easily removed by a water washing step before elution of the resin with ethanol. Ethanol eluted extract was concentrated under vacuum to obtain purified phenolics extract (LOH, 10.4 ± 0.4 g) with a yield around 2% (*w*/*w*) of dry weight of the extract.

Flavonoids and phenolic compounds have widely been described as producing a variety of biological effects. *Linum usitatissimum* harbors a panoply of bioactive compounds with potential pharmacological properties. Therefore, defining total phenolic content and concentration of flavonoids in LOH is a key step in the exploration of its therapeutic potential in the biomedical applications, especially tissue engineering nanocomposite scaffolds.

In the present research, the total amount of phenolics in LOH was determined according to the Folin–Ciocalteu procedure. The result showed that the LOH was found to contain a high number of phenols (740.06 ± 1.2 μg GAE/mg).

The total flavonoid content of LOH was determined by utilizing aluminum chloride colorimetric method. The tested LOH was found to contain high amounts of flavonoids (550 ± 2 μg RE/mg).

Phytochemical screening of LOH by means of UPLC-MS analysis revealed the presence primarily of phenolic acids and flavones *C*-glycosides compounds. A variety of these phenolic compounds were identified, and these relative proportions are different (data not shown). The results obtained were compared with those obtained by Tchoumtchoua et al. (2019) [27] and confirmed that the aerial parts of flax contain a significant source of phenolic compounds.

### 2.2. Synthesis of Hydrogel Nanocomposite

**Scheme 1:**
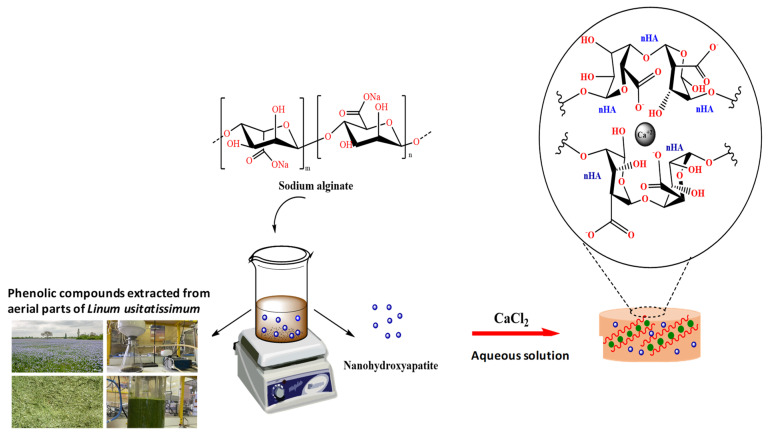
General scheme for synthesis route of the Alginate/nHA/LOH nanocomposite gels.

The detailed synthetic procedure of the alginate-based gel was sketched in Scheme 1. The main purpose of this study was to fabricate a bone-mimicking porous scaffold based on a highly biocompatible matrix, namely, Alg, which contains osteoconductive nHA nanoparticles and LOH. The LOH is a mixture of phenolic compounds extracted from aerial part of flax and was used as drug loaded into alginate-nHA nanocomposite hydrogel. The preparation method of nHA granules by wet chemical precipitation method used in this study was reported earlier [29]. The solid-gel is achieved by ionotropic gelation procedure by pouring the hydrogels into CaCl_2_ solution and then freeze-dried in −80 °C.

### 2.3. Characterization Studies

#### 2.3.1. Characterization of nHA

**Figure 1:**
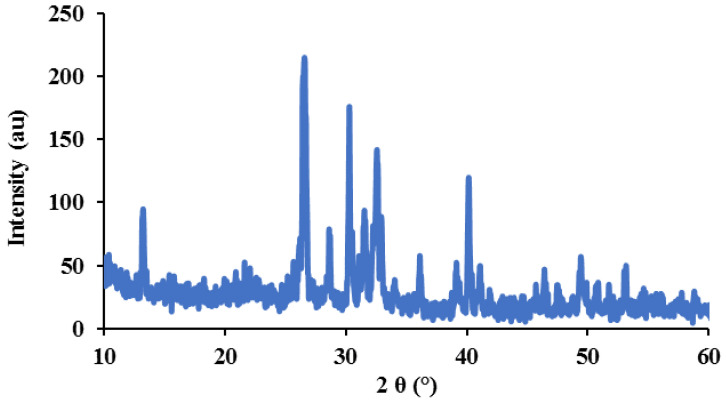
XRD pattern of the synthesized nHA.

The hydrodynamic size, Zeta potential, and the criticality of the synthesized nHA were characterized using XRD analysis, DLS, and Zetasizer, respectively. The powder XRD patterns of nHA are shown in Figure 1. As can be seen, the nHA nanoparticles revealed sharp and intense peaks at 2θ of 25.8°, 31.86°, 40.16°, and 49.6° that were indexed to (002), (211), (310), and (123) reflection planes [30,31]. The synthesized nHA exhibited XRD pattern similar to the standard HAp available from the Joint Committee on Powder Diffraction Standards (JCPDS; standard number 84-1998). The DLS measurements of nHA were carried out in deionized water between −200 and +200 mV. The nHA has a negative surface charge with a maximum peak at −9.41 mV with an average size of 391.3 nm and polydispersity index (PDI) of 0.287.

#### 2.3.2. Morphology and Microstructure of Hydrogel Nanocomposite

**Figure 2:**
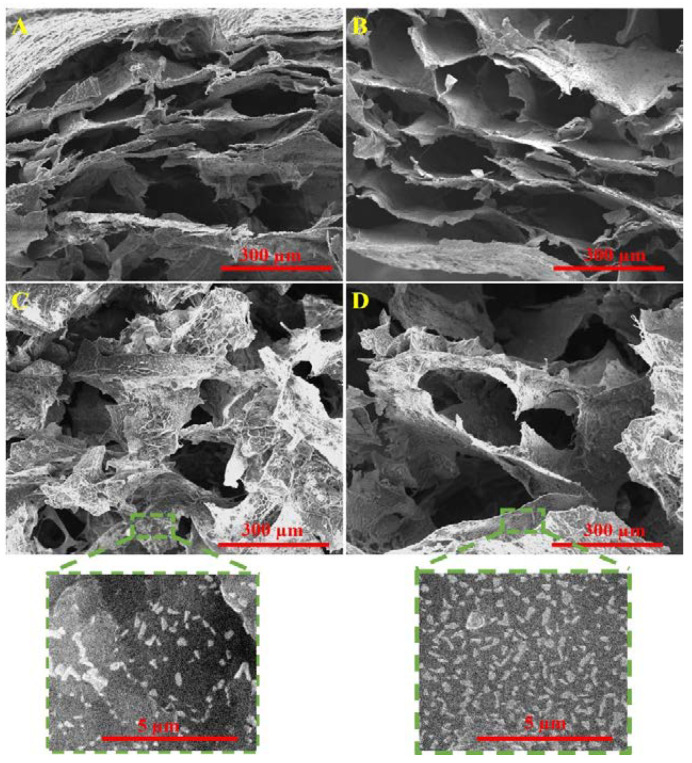
SEM micrographs of (**A**) pure Alg, and cross-linked Alg/LOH/nHA nanocomposite with different contents of nHA (**B**) 7% nHA, (**C**) 14% nHA, and (**D**) 21% nHA. High magnification SEM image of the cross section indicating the nHA crystals. The scale bars in Figures (**A**–**D**) are 300 μm, the scale bare in the magnified Figures are 5 μm.

To achieve a view of the structure of produced nanocomposite hydrogel, after freeze-drying, SEM micrographs of the scaffolds cross-sections and surfaces were provided (Figure 2A,B). The results showed that the fabricated hydrogel nanocomposites have a porous microstructure with interconnected pores. As a scaffold for tissue engineering, the nanocomposites have a sponge state and exhibit an open and interconnected macroporous architecture containing a lot of pores with irregular holes. The porosity (vol%) quantification was estimated within ranges of approximately 80–90%. The cell behaviors, including cell spreading and proliferation, transportation of nutrients and oxygen, waste products, and the ability of the scaffold to retain water are affected by porosity [32,33]. Therefore, LOH-loaded hydrogel with the pore size distribution of 100–200 µm can be useful for fabricating scaffold for cell attachment and growth or to incorporating biological substances. At higher magnifications of Figure 2C,D, nHA nanoparticles incorporated into the hydrogel with an average size of 50 nm, and plate-like shape and fine dispersibility can be seen.

#### 2.3.3. Elemental Analysis

**Figure 3:**
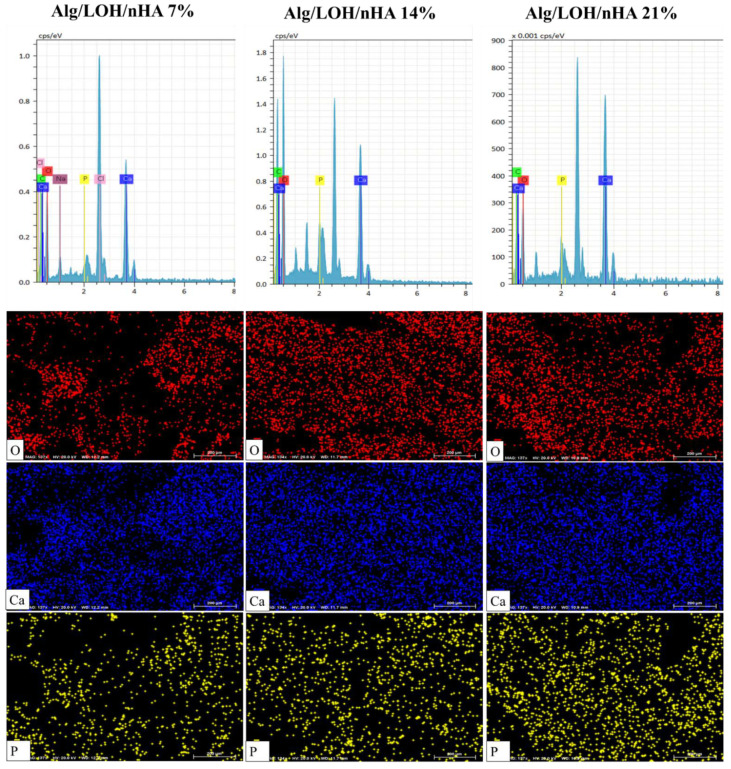
EDX spectrum of the Alg/LOH/nHA cross-linked polymer on double-sided adhesive carbon tape.

Semi-quantitative elemental analysis of the fabricated hydrogel nanocomposites was conducted using the EDX analysis and the results are presented in Figure 3. The spectrum approved the presence of calcium and phosphate elements while an increase in the content of Ca and P of the samples is evident with increasing nHA percentage. The calcium quantity obtained is related to both the Ca in nHA and the Ca^2+^ bridges linked to the alginate hydrogel.

#### 2.3.4. FTIR Spectroscopy

**Figure 4:**
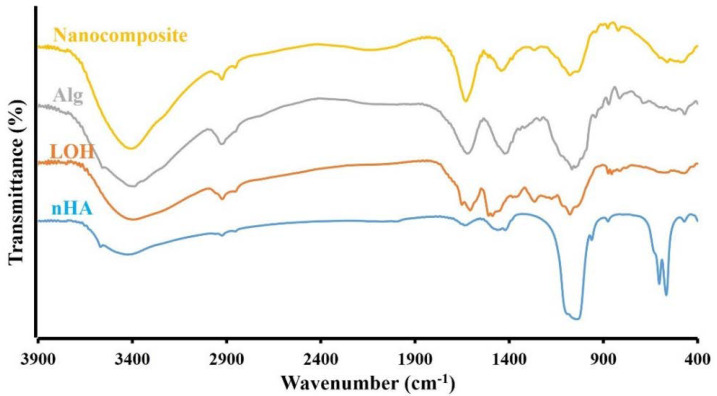
FTIR spectra of synthesized Alg/LOH/nHA nanocomposite compared to pure Alg, nHA, and LOH.

Molecular interactions between Alg polymer, nHA, and LOH polyphenolic extract in the Ca^2+^ cross-linked nanocomposites were examined by the Fourier transform infrared spectroscopy (FTIR). In Figure 4, FTIR spectroscopy of fabricated Alg/nHA/LOH was compared with FTIR peaks of the pure state of Alg, nHA and LOH. The FTIR spectra of pure Alg absorbance bands in 1614 and 1415 cm^−1^ correspond to the asymmetric and symmetric stretching vibration of C=O groups, respectively. The observed peaks in 1080 cm^−1^ and 1030 cm^−1^ are assigned to C–O–C bands vibrations [30,34,35,36]. The phosphate absorption bands in the FTIR spectrum of nHA are identified by peaks at 472.9, 560, 603.5, 962, 1029, and 1092 cm^‒1^ and two weak carbonate bands are observed at 1416 and 1456 cm^‒1^ for nHA. The stretching and flexural modes of the hydroxyl (OH^−^) group in the apatite lattice are confirmed by absorption bands at 3566, 1622, and around 630 cm^−1^ [31,36]. In LOH FTIR spectra, the C–H symmetrical and asymmetrical stretching vibration in CH_2_ and CH_3_ groups are revealed at around 2920 and 2848 cm^−1^, while their bending vibrations are located at 1487 and 1450 cm^−1^. The peaks at about 1650 cm^−1^ are related to carbonyl (C=O) groups and at near 1263, 1076, and 1030 cm^−1^ are due to C–O and C–C stretching vibrations [36,37,38]. FTIR spectrum of nanocomposite gel contains all absorbance peaks of Alg, LOH, and nHA, with some shifts and overlapping in the intensity and/or shape of the absorption peaks. The presence of LOH and nHA and the hydrogel formation through crosslinking have caused a modification in OH stretching vibration to a narrower peak. The spectra also showed an increase in the absorption vibrations of the C=O group from 1614 and 1415 cm^−1^ to 1624 and 1435 cm^−1^. The removal of the peaks related to the –CH_3_ and –CH_2_ bending of LOH at 1487 and 1450 cm^−1^ may be due to the hardening of the structure. It was mentioned that nHA has an absorption band at 1029 and 1092 cm^−1^ due to the stretching of PO_4_^3−^. Alg also has a strong absorption band at this place referred to C–O–C group [31,36]. Therefore, broadband centered around1047 cm^−1^ is contribute to the overlap of C–O–C stretching of Alg and PO_4_^3−^ stretching of nHA after the integration of Alg, nHA and LOH. The observed broadband at about 484 and 569 cm^−1^ is attributed to the PO_4_^3−^ bending vibrations of nHA. It could be concluded that the incorporation of nHA and LOH into Alg was successful.

### 2.4. Swelling Behavior

**Figure 5:**
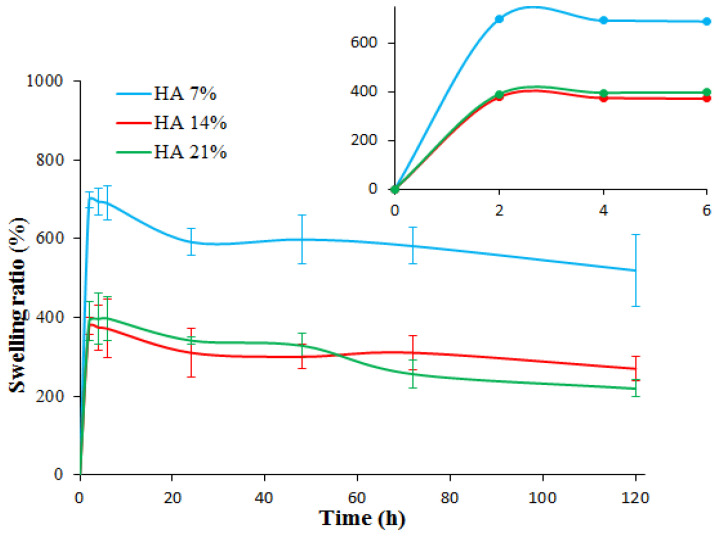
The effect of nHA content on the swelling rate of Alg/LOH/nHA nanocomposite hydrogel.

The swelling properties of hydrogels can reflect their water uptake capacity. The percentages of swelling at different time points are presented in Figure 5. The swelling behavior of Alg/LOH/nHA gel shows highly dependent on the amount of nHA nanoparticles. In the presence the swelling ratio decrease with the increasing of nHA content to 14% (*w*/*w*) and then increased again when the nanoparticle concentration increased to 21% (*w*/*w*). The swelling is dependent on the hydrophilic groups at the matter. With increasing nHA content, there are fewer hydrophilic groups available as they are bonded to the nHA. Besides that, the swelling of the hydrogels involves large-scale segmental motion, which ultimately results in an increased distance between the polymer chains. When there is a high distribution of nHA throughout the gel, the nHA particles could contract and limit the movability of Alg polymer chains, which all work together to develop low swelling of the gel [35,39,40]. A further increase in nHA concentration in the gel limits the transfer of sol–gel of Alg, so the nanocomposite hydrogel is cross-linked loosely, which facilitates the water permeation into the gel and increases their swelling [40,41,42].

### 2.5. LOH Entrapment Efficiency (EE) and Loading Capacity (LC)

The effect of different ratios of nHA (7%, 14%, and 21% *w*/*w* of total Alg) on the EE% and the LC% of LOH was evaluated and the results are presented in Table 1. For this purpose, the EE% and LC% parameters of LOH entrapped within all three of the hydrogel nanocomposites were determined spectrophotometrically (λ_max_ = 270 nm), deducting the amount of LOH that remained in the CaCl_2_ solution supernatant after crosslinking from the initial amount in the reaction solution. Hydrogel nanocomposites with a higher nHA ratio exhibit a higher quantity of EE% and higher loading efficiency values with a nonlinear trend. According to previous articles, nHA can enhance the loading and encapsulation of hydrophilic and anionic drugs through hydrogen bond formation and/or electrostatic interactions [40,42,43]. Loading of LOH in Alg/nHA composite could be attributed to twofold interactions of LOH with both nHA and alginate matrix.

### 2.6. Release Behavior of LOH

**Figure 6:**
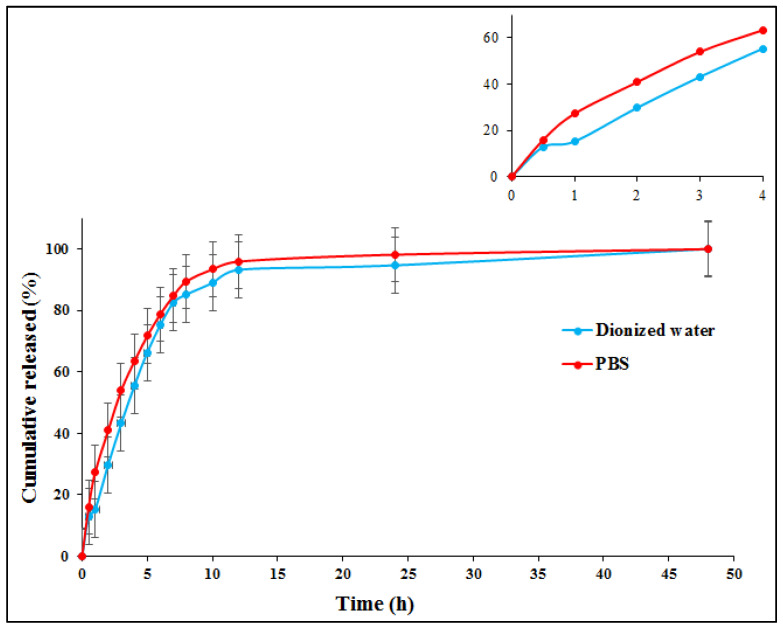
Cumulative release profiles of the Alg/LOH/nHA 21% nanocomposite gel in deionized water and pH 7.2 PBS.

We have tried to assess the cumulative release behavior of LOH from the fabricated hydrogel nanocomposite labeled with Alg/LOH/nHA 21% by UV spectroscopy at the physiological pH 7.2 in PBS and deionized water. Figure 6 demonstrates that 50% of LOH is released from the extract-loaded gel within the first 3 h of analysis, while in aqueous media, nanocomposite gel can release approximately 40% of extract during this time. Although the faster release was observed in PBS at all time points, accompanied by a relatively burst release in the early hours, most LOH was completely released from cross-linked composite gels in about 24 h in both media. These observations are related to the low stability of Alg-based hydrogels in physiological and aqueous environments because of the release of Ca^2+^ ions in the environment, resulting in breaking and reducing crosslink bonds [44,45,46]. The ion exchange reactions of phosphate and sodium ions with Ca^2+^ in PBS intensified the phenomenon [36,47,48].

### 2.7. In Vitro Results

#### 2.7.1. Hemocompatibility Assay

**Figure 7:**
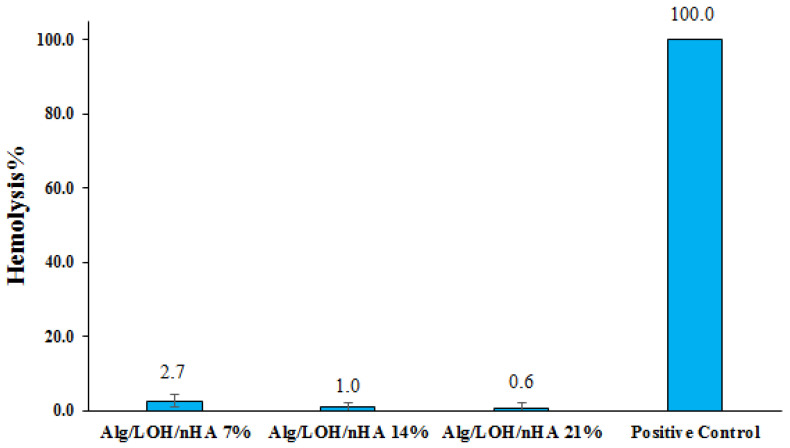
Assessment of hemolytic potential of the Alg/LOH/nHA in compared positive control after 1 h incubation with diluted blood with PBS. H_2_O and PBS are the positive and negative controls, respectively.

Hemocompatibility assessment was carried out to evaluate the disruption of red blood cells (RBC) membrane induced by a foreign substance that causes the leakage of the blood cell contents into the surrounding liquid [49,50,51]. It is established that hemolysis lower than 10% is acceptable and considered hemocompatible [52,53]. In this work, the hemolytic property of Alg/LOH/nHA was tested via direct contact of samples with RBC solution, and the results are shown in Figure 7, representing the calculated hemolysis percentage. In all cases, total hemoglobin concentration was within the reference range and no significant hemolysis was observed compared to the positive control. Moreover, the hemolysis percentage of the negative control group was calculated as 0%.

#### 2.7.2. Antioxidant Activities

**Figure 8:**
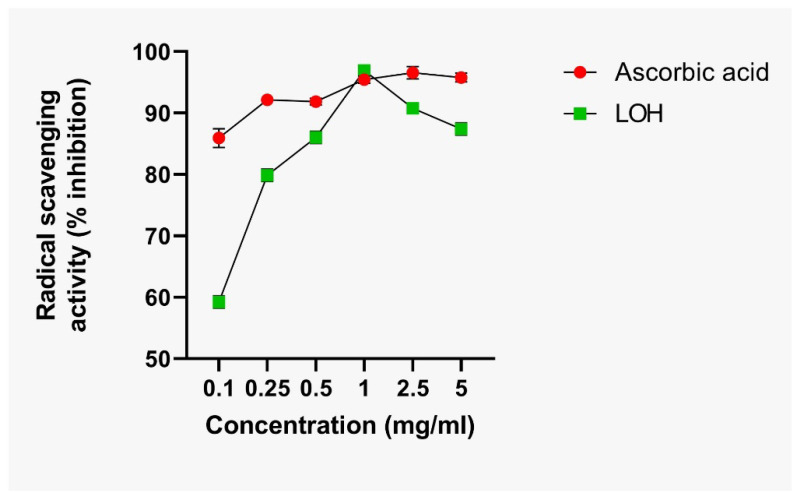
The antioxidant activity of LOH and ascorbic acid.

The antioxidant activity of LOH was assessed and compared with ascorbic acid (as the positive control) using the DPPH assay kit and the results are presented in Figure 8. The result showed significant antioxidant activity that was more than 95% similar to the ascorbic acid. The antioxidant substances can modulate the inflammatory responses induced after the injury [54].

#### 2.7.3. Cell Proliferation Results

**Figure 9:**
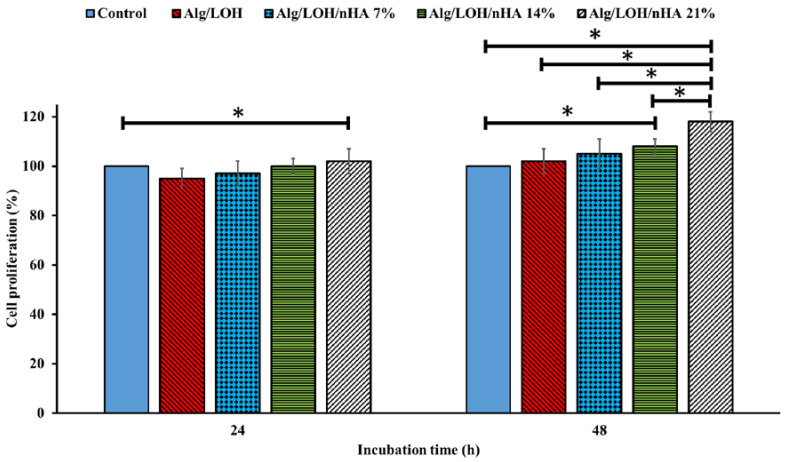
Proliferation of MG63 cells on the prepared nanocomposite hydrogels measured by the MTT assay. Mean ± SD, *: *p* < 0.05.

The cellular compatibility of prepared hydrogel nanocomposites was assessed on the MG-63 cell line using the MTT assay kit and the results are presented in Figure 9. The cells were cultured in contact with the nanocomposite gels, which served as a scaffold for tissue repair and evaluated for proliferation and survival of the cell. The materials’ toxicity level for cells was evaluated using MTT assay at 24 h and 48 h after initial seeding and results are shown in Figure 9. The osteoblast-like MG-63 cells indicated a good degree of proliferation after seeding onto all three types of scaffold compared with the control group. Figure 9 reports that the scaffold containing more nHA exhibits higher cell proliferation.

Based on the outcomes achieved from blood and cell compatibility evaluations, we can conclude that the fabricated hydrogel nanocomposites, as scaffolds are generally non-toxic and safe under in vitro conditions and can be suitable and promising materials for bone tissue engineering applications.

## 3. Discussion

The primary aim of this work was to introduce an alginate-based nanocomposite hydrogel containing purified extract obtained from aerial part of *Linum usitatissimum* (LOH) labeled with Alg/LOH/nHA and assessment of its potential as a porous scaffold for use in bone tissue engineering purposes. Given this, alginate was incorporated with nHA as inorganic reinforcing and an osteoconductive moiety of composite gel that appears particularly practical because of having excellent biocompatibility and osteoconductivity and strong mechanical properties [30,35,55]. Many articles have reported that sodium alginate forms porous, strong, and stable solid gels via ionically cross-linking with calcium [40,56,57,58,59]. Although the cross-linked alginate-ion gel is known as a biocompatible and bioactive network with unique cell adhesion properties, it has disadvantages, such as limited durability in physiological conditions, which affects the swelling behavior and release of the drug [44,45,46,59,60]. The prepared composite gel was loaded with LOH, an extract employed in diverse medicine for its biologically active compounds [36,37,61,62]. According to Wang et al., several intermolecular cross-links may be formed through Ca^2+^ between nHA and Alg, LOH and Alg, and nHA and LOH due to the divalent nature of Ca ions [35].

As noted, synthesized nHA are plate-like with an average size of 50 nm (according to the SEM images). After freeze-drying, Alg/LOH/nHA scaffold revealed a network morphology with porosity above 80–90% and interconnected pores (size 100–200 nm) on both cross-sections and surface, thus providing a suitable environment for the entry of oxygen and nutrients for the cultured cells and the exit of their waste products. Therefore, porous interconnected construction is a key characteristic for bone tissue engineering scaffolds. Cell adhesion, proliferation, and differentiation, as well as angiogenesis in the scaffold, are affected by pore size, porosity percent, and pore size distribution. The appropriate porosity percent also facilitates the release of the drug from the nanocomposite and increases its swelling and water absorption capacity [31,40,63,64].

Swelling behavior is a significant feature for scaffolds of bone tissue engineering, because the scaffold must remain stable in the biological environment until cells have reached a certain growth [31,40]. The swelling degree also has direct effects on the drug delivery and release. However, unlimited swelling leads to the deformation and loss of integrity and strength of the structure and tension in the surrounding tissues [35,39,41]. Swelling behavior is a characteristic dependent on the porosity and hydrophilicity of the polymer. Sodium alginate shows high hydrophilicity and porosity capacity, and thus, represents a superior swelling and water absorption percentage. After ion cross-linking, the gel shows decreased hydrophilicity, mobility, and swelling capacity [30,35,39,40,41,42]. In the current work, based on the SEM images, the proper and homogeneous distribution of nHA in the Ca-alginate matrix was evident. The superficial interactions between gel hydrophilic groups and nHA (14% *w*/*w*) reinforcements further reduce the swelling rate. High incorporation of nHA (21% *w*/*w*) into calcium alginate gel enhance the water permeation into the structure and swelling degree due to loosening the cross-links [35,42]. Accordingly, the swelling behavior of the nanocomposite gel can be controlled and optimized by the introduced content of inorganic reinforcing, here nHA.

LOH is an extremely hydrophilic and very water-soluble extract, and thus, it is not simply maintained in porous polymeric networks, such as Alg hydrogels [36]. On the other hand, nHA and sodium alginate are both very good biocompatible substances, but when each is used purely as a drug carrier, they have a burst release and the drug is washed away in a short time [35,40]. In the present instance, EE% and LC% of LOH in Alg/nHA organic-inorganic composites were on the rise when we increased the nHA content. This result could be related to the upward interaction of the LOH with the Alg/nHA nanocomposite [43]. Additionally, a sustained release of LOH from the nanocomposite gel was observed for about 12 h. The addition of nHA improved the loading of LOH in composite. They also showed acceptable blood compatibility and notable cells cytocompatibility towards MG-63 osteosarcoma cells. This observation can be partly due to the osteoconductive properties of nHA, which enhanced the cell adhesion on the hydrogel nanocomposite. Results of these tests showed promising performance for bone tissue regeneration and repair. Previous studies also showed that the incorporation of HA crystals enhances the proliferation of cells. Bendtsen et al. [65] fabricated Al-polyvinyl alcohol-HA hydrogel for 3D bioprinting bone tissue engineered scaffolds and observed high cell viability. In another study, Yan et al. [39] fabricated injectable Alg/HA gel scaffold for drug delivery and bone tissue engineering. The authors reported that the fabricated scaffold provided suitable biocompatibility due to the presence of HA crystals.

## 4. Materials and Methods

### 4.1. Materials

Sodium alginate (SA) and calcium chloride (CaCl_2_. 4H_2_O), used as a crosslinking agent of SA, were purchased from Merck (Darmstadt, Germany). The linseed plants (*Linum usitatissimum* L., winter flax species) were provided by the Linea industry (Wavignies, France). The plants were harvested from the fields during the early stages of flowering, in May 2020. After the sampling, the aerial part of the plants was lyophilized and then stored under vacuum at −20 °C. All used solvents were obtained from Sigma-Aldrich (St. Louis, MO, USA). The MTT assay kit was purchased from Roth (Karlsruhe, Germany). Fetal Bovine Serum (FBS), DMEM/F-12 cell culture medium, Trypsin-EDTA, and Penicillin-Streptomycin (Pen-Strep) were obtained from Gibco (Karlsruhe, Germany). All chemicals were used as received without any further purification, except the ones mentioned specifically. The MG-63 cell line was acquired from the Pasteur Institute, Iran. Plastics and tissue culture plates were from SPL, Korea.

### 4.2. Extraction, Purification, and Characterization of Extract from the Aerial Parts of Linum Usitatisimum

#### 4.2.1. Extraction and Purification

The plant material was ground using a blade crusher with 1 mm screens. Just before extraction, the powder was ground again from an electric mill to decrease particle size and improve extraction yields. The powder thus obtained was subjected to extraction for one hour at room temperature with agitation using an electric stirrer. In total, 500 g of powder were immersed in 10 L of an H_2_O/MeOH mixture (50/50, *v*/*v*). The solution obtained was filtered through a Büchner funnel and then evaporated under the vacuum at 40 °C in order to remove the methanol. The crude extract (4 L) was applied to the XAD 16-N resin. The resin was then washed with 2 L of H_2_O to remove non-recoverable compounds, and then eluted with 2 L of an 80/20% EtOH/H_2_O mixture in order to recover the interest compounds. This latter fraction, containing polyphenolic molecules, was then evaporated under vacuum, and then lyophilized to obtain the dry matter of the purified extract from the aerial part of *Linum usitatisimum* (LOH).

#### 4.2.2. Determination of the Total Phenolic Content

The Folin–Ciocalteu assay was used to quantify the total phenolic content. The test samples (1 mL) were mixed with sodium carbonate (2%, *w*/*v*) and the Folin–Ciocalteu phenol reagent (10%, *v*/*v*), and the mixture was allowed to stand for 10 min. The absorbance of the mixture was measured at 750 nm and a standard curve was prepared using gallic acid. The results were expressed as milligrams of gallic acid equivalents (GAE) per gram of the LOH

#### 4.2.3. Determination of Total Flavonoids Content

The sample was dissolved in 600 µL to obtain a concentration of 250 mg/L and 600 µL of AlCl_3_ (2%) was added. After 60 min, the absorbance was performed at 420 nm. The concentration of the total flavonoid content in the samples was calculated from the calibration curve performed with rutin (5 to 200 μg/mL) and expressed as rutin equivalent (RE) per gram of the LOH.

### 4.3. Preparation of the Nanocomposite Hydrogels

The nano hydroxyapatite (nHA) was produced through a wet chemical precipitation procedure, as described in the previous report [17]. Afterward, 20, 40, and 60 mg of nHA powder was added to 10 mL of LOH solution (1% *w*/*v*), which was prepared in distilled water. The nHA was dispersed under magnetic stirring and ultra-sonication to achieve the uniform distribution of nHA particles. A proper amount of Alg was added to the prepared solution to obtain the final concentration of 2% *w*/*v* and constantly stirred for 2 h. The construct was cross-linked with 2 M CaCl_2_ aqueous solution. Finally, the resulting hydrogels were washed several times with distilled water to remove unreacted Ca^2+^. The prepared composite materials were pre-frozen at −20 °C for 24 h to stabilize and then lyophilized under vacuum freeze-drying at −50 °C for 24 h. Finally, obtained LOH-loaded Alg/nHA nanocomposite gels were stored at 4 °C until use.

### 4.4. Spectral Characterization of the Synthesized Alg/nHA/LOH

FTIR analysis of Alg/nHA/LOH samples, sodium alginate, and nHA powder was carried out by accumulating 16 scans at a resolution of 4 cm^−1^ over a wavenumber region of 400–4000 cm^−1^ using an FTIR spectrometer (Spectrum RX1 FTIR system, Perkin-Elmer, Texas City, TX, USA). The Powder X-ray diffraction (XRD) characteristics of the nHA were carried out using a Philips Xpert instrument. X-ray diffraction data were collected using Cu-Kα radiation operating at 40 kV and 30 mA with 2θ ranging from 10 to 90° at a scan speed of 0.08 s^‒1^. For each sample, peak intensity changes were analyzed relative to the reference band within the same spectra. The particle size, polydispersity index (PDI), and zeta potentials of nHA and synthesized samples were measured using a dynamic light scattering (DLS) instrument (Zetasizer Nano ZS90, Malvern, Worcestershire, UK). All the measurements were conducted in triplicate.

### 4.5. Morphological and Structural Analyses

The surface and cross-section morphology of lyophilized hydrogel nanocomposites were visualized and analyzed with a Scanning Electron Microscopy (SEM, Philips XL-30, Eindhoven, The Netherlands). The samples were sputter-coated with a thin layer of gold and scanned at an accelerating voltage of 20 kV at different magnifications. Energy-dispersive X-ray (EDX) was also used for elemental mapping and analyzing the chemical composition of samples.

### 4.6. Swelling Studies

The swelling behavior of the fabricated Alg/nHA/LOH aerogels was evaluated for samples with different nHA concentrations and each test was carried out in triplicate. The analysis was carried out by measuring the weight of prepared hydrogel in a dry (W_dry_) and a wet (W_wet_) state as previously described. The studies were performed for 7 days through continuous immersion of specimens in media. Then, Equation (1) was used to calculate the percentage of the swelling ratio or the water adsorption of Alg/nHA/LOH:

(1) $$\mathrm{Swelling}\;\mathrm{ratio}\;\left(\%\right)=\frac{W_{\mathrm{wet}}-{\;W}_{\mathrm{dry}}}{W_{\mathrm{dry}}}\times 100\;$$

### 4.7. Entrapment Efficiency (EE) and Loading Capacity (LC)

The entrapment of the LOH into the structure of the hydrogel nanocomposite was carried out during the cross-linking process. For entrapment efficacy% (EE%) measurement the absorbance of the supernatant was recorded using UV-VIS spectrometer at λ_max_ (270 nm) of LOH. A stock solution of 0.1 mg/mL LOH in distilled water was diluted to the desired concentrations to draw a calibration curve and the concentrations obtained from it was employed to calculate the EE% and loading capacity (LC) according to Equations (2) and (3):

(2) $$\mathrm{EE}\;\left(\%\right)=\frac{\left(W_{t}-{\;W}_{s}\right)}{W_{t}}\times 100$$

(3) $$\mathrm{LC}\;\left(\%\right)=\frac{\left(W_{t}-{\;W}_{s}\right)}{W_{\mathrm{np}}}\times 100$$

where W_t_ is the initial amount of LOH dissolved in reaction solution, W_s_ is concentration of released LOH in the supernatant after gelation, and W_np_ is the mass of the final dried Alg/nHA/LOH.

### 4.8. Release Study

The release kinetics of LOH from hydrogel in different releasing mediums of PBS and deionized water were compared. In any solution, certain quantities of formulated gels were immersed in a 20 mL releasing medium at room temperature. The release rate of the LOH from Alg/nHA/LOH 21% was determined periodically via withdrawing 2 mL of supernatant solution and depositing it into an Eppendorf tube at appropriate time intervals up to 2 h. Two ml of fresh releasing media was added at each time point to maintain the solution volumes in 20 mL and the sink condition. Cumulative release of LOH in the aliquots was quantified by measuring the absorption band centered at 270 nm by UV−VIS spectroscopy and calibration curve axis. The data represent triplicate determinations.

### 4.9. In Vitro Evaluations

#### 4.9.1. Hemolysis Assay

For the hemocompatibility assay, the blood was obtained from a healthy volunteer human and the operation method was according to previous reports [39]. Briefly, 2 mL of fresh anticoagulated blood was added to 2.5 mL PBS. The diluted blood (150 μL) was treated with the samples for 1 h at 37 °C. Diluted blood with PBS (0% lysis) and deionized water (100% lysis) served as negative and positive controls, respectively. After the incubation, the samples were centrifuged for 5 min at 1500 rpm to collect plasma. The hemolytic potential of hydrogels was assayed by measuring the absorption of released hemoglobin in plasma at 545 nm on a Microplate Reader. Each experiment was repeated in triplicate for each test solution. Hemolysis percent was obtained by Equation (4):

(4) $$\mathrm{Hemolysis}\;\left(\%\right)=\frac{D_{t}-D_{\mathrm{nc}}}{D_{\mathrm{pc}}-{\;D}_{\mathrm{nc}}}\times 100$$

where D_t_ is the absorbance value of the sample at 545 nm, D_nc_ is the absorbance of the negative control, and D_pc_ is the absorbance of the positive control.

#### 4.9.2. Antioxidant Activities Measurement

Different concentrations (0.1, 0.25, 0.5, 1, and 2.5 mg/mL) of the samples were prepared. In total, 50 µL of the sample with different concentrations was mixed with a 100 µL methanol solution of DPPH (100 µM) in a 96-well plate. After placing in the dark for 40 min at room temperature, the absorbance was noted at 517 nm and normalized to a blank sample (the blank contained sample dilution + methanol) and the control sample (water + DPPH solution). Equation (5) was applied to calculate the inhibition percentage:

(5) $$Inhibition\;\%=1-\frac{\mathrm{Abs}\left(\mathrm{sample}\right)-Abs\left(blank\right)}{Abs\left(control\right)-Abs\left(blank\right)}\times 100$$

#### 4.9.3. Cell Proliferation Measurement

For the in vitro biocompatibility evaluation, the appropriate portions of Alg/nHA/LOH aerogels were placed into the 96-well polystyrene plate, sterilized by immersion in ethanol (70%) for 4 h at room temperature, and washed by sterilized PBS three times before cell culture. The MG-63 cells were used as the model cell to investigate the effects of the synthesized gel on cell survival. A cell suspension of MG-63 cells was seeded on each of the three types of gels at a density of 1 × 10^4^ cell/well and left at 37 °C in 95% humidified air and 5% CO_2_ overnight to attach to the substrates. Cell media composed of DMEM with 10% FBS and 1% penicillin/streptomycin was added to each well. The culture medium was changed into the fresh one every day, and the cells were cultured for 48 h. The number of viable cells was determined by measuring their mitochondrial reductase activity using the tetrazolium-based colorimetric method (MTT conversion test). This assay depended on the cellular reductive capacity to metabolize the yellow tetrazolium salt, 3-(4,5-dimethylthiazol-2-yl)-3,5-diphenyl tetrazolium dye (MTT), to a highly colored Formazan product. The viability of the cultured cells was evaluated by adding 100 μL of MTT to any well after 48 h and incubated at 37 °C for 4 h. The medium was withdrawn and 200 μL DMSO was added to each well and agitated on a shaker thoroughly to dissolve the Formazan crystals. The UV absorbance of the Formazan solution was measured spectrophotometric ally at 570 nm by the Microplate Reader.

### 4.10. Statistical Analysis

All experiments were run in triplicate per sample. Quantitative data were reported as mean ±standard deviation (SD). Statistical analysis was performed using a one-way analysis of variance. Comparison between means was done using the t-test with a minimum confidence level of *p* ≤ 0.05 for the statistical significance using R-3.3.3 software.

## 5. Conclusions

Nanotechnology as an enabling technology has unprecedented effects on different fields of biomedicine. Nanostructured bone tissue engineering scaffolds have shown promising results in accelerating and improving the bone healing process. In the current study, we fabricated a 3D hydrogel nanocomposite based on Alg/nHA/LOH as the bone tissue engineering scaffold. The characterization results revealed that the fabricated 3D nanocomposite scaffolds have a porous microstructure with interconnected pores architecture and nHA crystals are dispersed throughout the scaffold. The in vitro evaluations showed that the fabricated nanocomposite scaffolds are hemocompatible and cytocompatible with beneficial proliferative effects. Moreover, the incorporation of LOH imparted antioxidant activity to the scaffold. The results of the current study indicated that the produced 3D nanocomposite scaffolds have beneficial physicochemical and biological properties and can be applied as the bone tissue engineering scaffold.

## Figures and Tables

**Table 1:** The EE% and LC% of LOH-loaded cross-linked hydrogel for LOH.

Group	Entrapment Efficacy (%)	Loading Capacity (%)
Alg/LOH/nHA 7%	55.07	1.47
Alg/LOH/nHA 14%	61.47	1.71
Alg/LOH/nHA 21%	63.66	1.80

## Data Availability

Further data are available on reasonable request from the corresponding author.
